# Prognostic value of mean velocity at the pulmonary artery estimated by cardiovascular magnetic resonance as a prognostic predictor in a cohort of patients with new-onset heart failure with reduced ejection fraction

**DOI:** 10.1186/s12968-020-00621-3

**Published:** 2020-04-30

**Authors:** Blanca Trejo-Velasco, Óscar Fabregat-Andrés, Pilar M. García-González, Diana C. Perdomo-Londoño, Andrés M. Cubillos-Arango, Mónica I. Ferrando-Beltrán, Joaquina Belchi-Navarro, José L. Pérez-Boscá, Rafael Payá-Serrano, Francisco Ridocci-Soriano

**Affiliations:** 1 Cardiology Department, Hospital Clínico de Salamanca, Instituto de Investigación Biomédica de Salamanca (IBSAL), Paseo San Vicente 182, 37007 Salamanca, Spain; 2 Cardiology Department, Hospital IMED, Avenida de la Ilustración, 1, 46100 Burjassot, Valencia Spain; 3 Unidad de Imagen Cardioresonancia Magnética, Centro Médico ERESA, Carrer del Marqués de Sant Joan 6, 46015 Valencia, Spain; 4 Cardiology Department, Hospital General Universitario de Valencia, Avenida Tres Creus 2, 46014 Valencia, Spain

**Keywords:** HFrEF, Cardiac MRI, Right ventricle, Ventricular-arterial coupling, Prognosis, pulmonary hypertension

## Abstract

**Background:**

Pulmonary hypertension (PH) conveys a worse prognosis in heart failure (HF), in particular when right ventricular (RV) dysfunction ensues. Cardiovascular magnetic resonance (CMR) non-invasively estimates pulmonary vascular resistance (PVR), which has shown prognostic value in HF. Importantly, RV to pulmonary artery (PA) coupling is altered early in HF, before significant rise in PV resistance occurs. The aim of this study was to assess the prognostic value of mean velocity at the pulmonary artery (mvPA), a novel non-invasive parameter determined by CMR, in HF with reduced ejection fraction (HFrEF) with and without associated PH.

**Methods:**

Prospective inclusion of 238 patients admitted for new-onset HFrEF. MvPA was measured with CMR during index admission. The primary endpoint was defined as a composite of HF readmissions and all-cause mortality.

**Results:**

During a median follow-up of 25 months, 91 patients presented with the primary endpoint. Optimal cut-off value of mvPA calculated by the receiver operator curve for the prediction of the primary endpoint was 9 cm/s. The primary endpoint occurred more frequently in patients with mvPA≤9 cm/s, as indicated by Kaplan-Meier survival curves; Log Rank 16.0, *p* <  0.001. Importantly, mvPA maintained its prognostic value regardless of RV function and also when considering mortality and HF readmissions separately. On Cox proportional hazard analysis, reduced mvPA≤9 cm/s emerged as an independent prognostic marker, together with NYHA III-IV/IV class, stage 3–4 renal failure and ischemic cardiomyopathy.

**Conclusions:**

In our HFrEF cohort, mvPA emerged as an independent prognostic indicator independent of RV function, allowing identification of a higher-risk population before structural damage onset. Moreover, mvPA emerged as a surrogate marker of the RV-PA unit coupling status.

## Background

Pulmonary hypertension (PH) is a frequent comorbid condition associated with heart failure (HF) [1], which implies a worse prognosis [2], in particular when right ventricular (RV) dysfunction ensues [3]. Although right heart catheterisation (RHC) is the gold standard technique for PH diagnosis, it entails certain risk of peri-procedural complications as well as radiation exposure. As a result, there is growing interest in PH evaluation by non-invasive procedures such as echocardiography and cardiovascular magnetic resonance (CMR) [4, 5].

CMR is an especially attractive diagnostic tool in this setting, as it provides accurate structural and functional assessment of the cardiac chambers –in particular the RV, which plays a determinant role in the prognosis of PH and HF [6]. CMR also assesses other parameters of the pulmonary circulation such as pulmonary artery (PA) pulsatility and mean velocity at the pulmonary artery (mvPA), which correlate strongly with mean pulmonary artery pressure (mPAP) in PH^−^ [7].

In recent years, increasing evidence supporting a comprehensive evaluation of the right ventricular-pulmonary artery (RV-PA) unit that integrates RV function and its adaptation to loading conditions is emerging [8, 9]. Importantly, RV-PA coupling not only encompasses the static component of RV afterload, expressed by pulmonary vascular resistance (PVR) [10, 11], but also its pulsatile element, which is altered at earlier disease stages in HF [12–14]. Accordingly, inefficient RV-PA coupling can be detected promptly and acts as a reliable prognostic indicator [15, 16].

In clinical practice, several RV-PA coupling indicators such as CMR derived E_max_/Ea ratio, tricuspid annular plane systolic excursion (TAPSE) to systolic PA pressure (sPAP) and PA stiffness and compliance are employed to stratify prognosis in PH and HF patients [15, 17–19], as direct measurement of end-systolic elastance (Emax, index of contractility) and PA effective elastance (Ea, index of arterial load) to calculate RV-PA coupling is complex and requires an invasive assessment of the right heart chambers to construct pressure–volume loops [8].

Recently, prognostic value of mvPA has been described in in a small sample of patients with HF with reduced (HFrEF) and intermediate ejection fraction (HFmEF) [20]. In this study we assessed the prognostic value of mvPA in a cohort of patients with new-onset HFrEF with and without associated PH. In addition, we evaluated the potential role of mvPA as a surrogate marker of the RV-PA unit coupling state.

## Methods

### Study population

Between January 2013 and January 2017, 238 consecutive patients (64.1 ± 12.6 years, 72% male) were prospectively included during their admission for acute new-onset HFrEF in the Cardiology Deptartment of a tertiary care hospital. Seventy patients included in a prior publication investigating mvPA in HF were also included in the current sample [20].

All patients underwent a CMR during index admission. Twelve patients with severe valvular heart disease, nine unable to undergo a CMR and seven lost to follow-up during the first 6 months after discharge were excluded leaving a final sample size of 210 patients. The study protocol complied with the Helsinki Declaration, the Institutional Review Board approved the study and all individuals consented for the procedures.

### Clinical variables

Demographic and clinical baseline variables were collected in all patients as were relevant analytical values including estimated glomerular filtration rate (eGFR) and N-terminal pro-brain natriuretic peptide (NT-proBNP), which were determined on admission. Significant electrocardiographic (ECG) parameters such as atrial fibrillation (AF) and underlying left bundle branch block were also recorded.

### Transthoracic echocardiography

Echocardiographic parameters such as left ventricular (LV) ejection fraction (LVEF), LV end-diastolic and end-systolic diameters; RV end-diastolic diameter on paraesternal long-axis, TAPSE and sPAP, as well as the RV-PA coupling indicator TAPSE/SPAP ratio were determined.

### Cardiovascular magnetic resonance

CMR was performed with a 1.5 T CMR system (Magneton Sonata, Siemens Healthineers, Erlangen, Germany) during the first week of admission, after clinical stabilization and compensation of HF, in euvolemic patients. Adequate control of ventricular response rate was performed in patients in AF prior to undergoing CMR. For cine imaging, breath-holding ECG-gated balanced steady-state free precession (bSSFP) sequences were used to acquire long- and short-axis slices and hence evaluate ventricular volumes and function. Short-axis slices were used to calculate ejection fraction and ventricular volumes using Simpson’s method. In AF patients presenting with significant variability in ventricular response rate, prospective acquisition of cine-imaging selecting a cycle-length shorter than the smallest R-R interval of the patient was performed for measurement of ventricular volumes and function [21].

Areas of late gadolinium enhancement (LGE), acquired after intravenous injection(0.15 mL/kg) of dimeglumine gadobenate 0.5 M, were assessed using inversion-recovery bSSFP sequences, 10 min after contrast administration adjusting the inversion time to null normal myocardium. A CMR expert (PM. G, MD) blinded to hemodynamic and echocardiographic data, visually identified myocardial LGE.

Flow imaging was performed perpendicular to the PA trunk with a velocity-encoded gradient echo sequence. In AF, flow imaging was averaged over several consecutive cardiac cycles and patients were asked to perform superficial breathing whenever breath-holding technique was not feasible. Images were analyzed using specific software (Argus, Siemens Healthineers), which automatically calculated mvPA as the integral of velocity in each of the voxels included within the PA contour over time. PA cross-section was outlined in each cardiac phase to estimate PA area and flow and calculate mvPA during the complete cardiac cycle and to determine minimum and maximum PA areas. PA pulsatility was calculated by means of the following formula = (maximum PA area-minimum PA area)/ minimum PA area × 100, validated in PH patients [7]. We estimated PVR by using the equation previously tested and validated: PVR [Wood Units (WU)] =19.38-[4.62 × Ln mvPA (cm/s)]–[0.08 × RV ejection fraction (RVEF)(%)] [22]. Furthermore, ventricular-vascular coupling ratio, comprised of RV maximal end-systolic elastance (Emax, index of contractility) and PA effective elastance (Ea, index of arterial load), was estimated through the following formula: Emax/Ea=stroke volume(SV)/end-systolic volume(ESV), validated by a prior study as an index of the RV-PA coupling state [23]. SV and ESV were determined through cine contours by CMR.

### Coronary angiography

X-ray coronary angiography was performed in patients with associated angina, regional wall motion abnormalities on echocardiography or a subendocardial LGE pattern identified by CMR suggestive of underlying ischemic heart disease*.*

The underlying etiology of cardiomyopathy was deemed ischemic when one of the following criteria was met [24]: history of myocardial infarction, ≥75% stenosis of left main or proximal left anterior descending artery or ≥ 75% stenosis of two or more epicardial vessels.

Clinically driven RHC was performed in a small subset of the total sample, at the discretion of the patient’s physician. RHC was performed within 48 h of CMR examination in all but four cases, in which both tests were fulfilled less than 96 h apart. RHC was performed with a PA catheter via femoral or brachial vein approach, employing standard methodology. Hemodynamic measurements included mean right atrial pressure, mPAP, pulmonary capillary wedge pressure (PCWP), cardiac output, PVR, pulse pressure and transpulmonary pressure gradient (TPG). PA compliance was calculated as the ratio RV stroke volume/PA pulse pressure, measured through CMR and RHC respectively [19].

### Clinical follow-up

Readmissions for HF and all-cause mortality were considered as major cardiovascular adverse events at follow-up and their combination was defined as the primary combined endpoint. Secondary endpoints consisted of the occurrence of each of these events separately. Events were prospectively recorded and physician-adjudicated through electronic health record review in all patients. Maximal follow-up length was established at 40 months.

### Statistical analysis

Patients were divided in two groups according to the optimal cut-off value of mvPA to predict the primary endpoint at follow-up. Cut-off value was calculated by receiver operating characteristic (ROC) sensitivity/1-specificity curve as the value attaining a largest area under the curve (AUC). Normal distribution of variables in both groups was confirmed with the Kolmogorov-Smirnoff test. Comparisons between both groups were made by X^2^ test or unpaired Student’s T-test, as appropriate. A multivariate Cox regression model was performed including all variables with a *P*-value of 0.10 in the univariate analysis, after exclusion of colinearity. Survival curves for mvPA were constructed with the Kaplan–Meier method and compared by means of the Log-Rank test. Linear correlation analysis was performed using Pearson coefficient. All tests were two-tailed, and a *P*-value≤0.05 was considered statistically significant. Statistical analyses were performed using SPSS for Windows (v.21.0 Statistical Package for the Social Sciences, International Business Machines, Inc., Armonk, New York, USA).

## Results

The studied sample included 210 patients with a first diagnose of acutely decompensated HFrEF requiring hospital admission. Prevalence of AF and history of previous coronary artery disease undergoing revascularization were 29.5 and 25.4% respectively, CMR LVEF was 26.9 ± 10.0%, Table 1. Etiology of LV dysfunction was non-ischemic in 134 patients (64.4%) including 56 cases of idiopathic cardiomyopathy (26.9%) and 25 patients (11.9%) with a final diagnose of tachycardiomyopathy, established in those cases where LV dysfunction was related to sustained tachyarrhythmia and partially or completely reversible after heart rate normalization [25]. Over half of the patients, *n* = 117(55.7%) were on NYHA II functional class on follow-up. Medical treatment at discharge was optimized in the majority of patients as follows: beta-blockers *n* = 197(93.8%), ACE inhibitors or angiotensin-2 receptor blockers *n* = 183(87.1%), mineralocorticoid antagonists *n* = 154(73.3%), diuretics *n* = 206(98.0%) and ivabradine *n* = 36 (17.14%).

**Table 1:** **Table 1** Baseline characteristics according to the primary combined endpoint

	No event *n* = 119	Primary combined endpoint *n* = 91	Total sample *n* = 210	*p*-value
Age (years)	62.9 ± 12.1	65.5 ± 13.3	64.1 ± 12.6	0.146
Sex, male(n,%)	84(70.6%)	63(69.2%)	147(70%)	0.832
Arterial hypertension(n,%)	76(63.9%)	60(67.4%)	136(65.4%)	0.594
Diabetes mellitus(n,%)	42(35.3%)	44(49.4%)	86(41.3%)	0.040
Dyslipidemia(n,%)	55(46.2%)	47(52.8%)	102(49%)	0.347
Atrial fibrillation(n,%)	32(26.9%)	30(33.0%)	62(29.5%)	0.339
Left bundle branch block(n,%)	31(26.5%)	18(21.2%)	49(24.3%)	0.384
Implanted cardiodefibrillator,(n,%)	20(17.1%)	24(27.6%)	44(21.6%)	0.302
Cardiac resynchronization therapy, (n,%)	8(6.7%)	2(2.2%)	10(4.8%)	0.114
Previous coronary artery disease(n,%)	25(21.0%)	28(31.1%)	53(25.4%)	0.096
eGFR (ml/min/1.73m^2^)	76.2 ± 16.1	69.2 ± 19.7	73.7 ± 17.9	0.012
Stage 3–4 Renal Failure (eGFR< 50 ml/min/1.73m^2^)	9(8.5%)	13(17.1%)	22(12.1%)	0.079
Sodium (mEq/L)	138.1 ± 2.7	137.3 ± 4.1	137.7 ± 3.3	0.128
Nt-proBNP (pg/mL)	5888 ± 4703	6043 ± 5041	5953 ± 4821	0.881
NYHA Functional Class				< 0.001
I	26(21.8%)	7(7.7%)	33(15.7%)	
II	73(61.3%)	44(48.4%)	117(55.7%)	
III	16(13.4%)	31(34.1%)	47(22.4%)	
IV	4(3.4%)	9(9.9%)	13(6.2%)	
Etiology of left ventricular dysfunction (n,%)				0.006
Ischemic	33 (27.7%)	41 (46.1%)	74(35.6%)	
Non-ischemic	86 (72.3%)	48 (53.9%)	134(64.4%)	
Idiopathic	38(31.9%)	18(20.2%)	56(26.9%)	
Tachycardiomyopathy	18(15.1%)	7(7.7%)	25(11.9%)	
LBBB	10(8.4%)	6(6.7%)	16(7.7%)	
Alcoholic cardiomyopathy	8(6.7%)	6(6.6%)	14(6.7%)	
Cardiotoxicity	5(4.2%)	3(3.3%)	8(3.8%)	
Infiltrative cardiomyopathy	2(1.7%)	5(5.5%)	7(3.3%)	
Non-compacted cardiomyopathy	4(3.4%)	2(2.2%)	6(2.9%)	
Myocarditis	1(1%)	1(1%)	2(1%)	
Echocardiography				
LVEF (%)	27.9 ± 9.3	27.5 ± 9.9	27.8 ± 9.6	0.712
LVEDD (mm)	59.6 ± 7.6	58.9 ± 7.4	59.3 ± 7.5	0.632
LVESD (mm)	46.8 ± 8.9	48.4 ± 9.2	47.6 ± 9.1	0.292
RV diameter (mm)	23.3 ± 5.9	24.7 ± 8.2	23.9 ± 6.9	0.270
SPAP (mmHg)^a^	46.8 ± 14.7	49.7 ± 14.3	48.1 ± 14.5	0.244
TAPSE (mm)	17.2 ± 3.8	16.4 ± 5.9	16.9 ± 4.7	0.370
TAPSE/SPAP ratio (mm/mmHg)^a^	0.42 ± 0.19	0.35 ± 0.25	0.39 ± 0.22	0.129
CMR				
LVEF(%)	27.5 ± 10.4	25.9 ± 9.5	26.9 ± 10.0	0.265
LVEDVI (mL/m^2^)	123.8 ± 39.1	137.3 ± 47.3	129.7 ± 43.2	0.025
LVESVI (mL/m^2^)	91.6 ± 37.7	103.3 ± 46.1	96.7 ± 41.8	0.050
RVEF(%)	42.4 ± 15.5	44.0 ± 15.9	43.1 ± 15.7	0.450
RVEDVI (mL/m^2^)	76.3 ± 31.5	78.0 ± 33.9	77.1 ± 32.5	0.705
RVESVI (mL/m^2^)	45.6 ± 26.0	46.2 ± 30.3	45.9 ± 27.9	0.863
LGE ischemic-pattern (n,%)	27(26.5%)	28(41.8%)	55(32.5%)	0.038
LGE non-ischemic pattern (n,%)	46(45.5%)	22(33.3%)	68(40.7%)	0.166
MvPA (cm/s)	10.9 ± 4.2	8.9 ± 3.2	10.1 ± 3.9	< 0.001
PVR (WU)	5.1 ± 2.4	5.8 ± 2.4	5.5 ± 2.4	< 0.036
RV-PA unit parameters				
Maximal PA-area (cm^2^)	8.2 ± 2.4	9.1 ± 2.2	8.6 ± 2.4	0.013
Minimal PA-area (cm^2^)	6.2 ± 2.4	7.1 ± 1.9	6.6 ± 2.1	0.010
PA pulsatility (%)	0.35 ± 0.20	0.30 ± 0.14	0.33 ± 0.18	0.102
RV Emax/Ea	0.89 ± 0.60	0.93 ± 0.62	0.91 ± 0.61	0.622
Right heart catheterisation parameters				
	**No event** ***n*** **= 35**	**Primary combined endpoint** ***n*** **= 20**	**Total sample** ***n*** **= 55**	**p-value**
MPAP (mmHg)	29.5 ± 10.7	34.8 ± 9.7	31.4 ± 10.5	0.077
PCWP (mmHg)	20.9 ± 8.2	20.6 ± 7.6	20.8 ± 7.8	0.904
RA pressure (mmHg)	11.9 ± 6.9	12.8 ± 5.7	12.3 ± 6.4	0.694
Cardiac output(l/min)	3.9 ± 1.5	3.1 ± 1.2	3.6 ± 1.4	0.090
PVR (WU)	2.7 ± 1.9	4.6 ± 2.8	3.4 ± 2.4	0.013
TPG (mmHg)	8.6 ± 6.0	13.8 ± 8.6	10.6 ± 7.4	0.013
Pulse pressure (mmHg)	23.7 ± 10.0	28.3 ± 15.2	25.4 ± 12.2	0.206
PA Compliance (mm^3^/mmHg)	1.87 ± 0.91	1.41 ± 0.84	1.67 ± 0.90	0.099

^a^ Measures available for 139 patients

*CMR* cardiovascular magnetic resonance. *CRT* cardiac resynchronization therapy. *Ea* effective elastance. *Emax* right ventricular maximal end-systolic elastance. *eGFR* estimated glomerular filtration rate. *ICD* implanted cardiodefibrillator. *LGE* late gadolinium enhancement. *LVEF* left ventricular ejection fraction. *LVEDD* left ventricular end-diastolic diameter. *LVESD* left ventricular end-systolic diameter. *mPAP* mean pulmonary artery pressure. *mvPA* mean velocity at the pulmonary artery. *Nt-proBNP* N-terminal brain natriuretic type peptide. *NYHA* New York Heart Association. *sPAP* systolic pulmonary artery pressure. *PA* pulmonary artery. *PCWP* pulmonary capillary wedge pressure. *PVR* pulmonary vascular resistance. *RA* right atrium. *RVEF* right ventricular ejection fraction. *TAPSE* tricuspid annular plane excursion. *TPG* transpulmonary gradient. *WU* wood units

During a median follow-up of 25 months (interquartile range 26 months), 91 patients met the primary endpoint at the expense of 70 HF hospitalizations and 49 all-cause deaths. Twenty eight of the patients that died had been previously admitted for HF.

Cardiovascular events presented more frequently in patients with diabetes mellitus, lower eGFR, New York Heart Association (NYHA) III-IV/IV functional class and ischemic cardiomyopathy, Table 1. Imaging parameters associated with excess event rates included increased maximal and minimal PA areas, greater PVR estimated by CMR and lower mvPA values. Enlarged LV volumes and LGE ischemic pattern were also more frequent among patients with cardiovascular events.

RHC was performed in 55(25.7%) patients of the total sample. In this subset of patients, hemodynamic parameters evaluating the presence of pulmonary vascular remodeling such as PVR and TPG displayed higher values in patients that met the primary combined endpoint, Table 1.

### Baseline patient characteristics according to mvPA

**Fig. 1:**
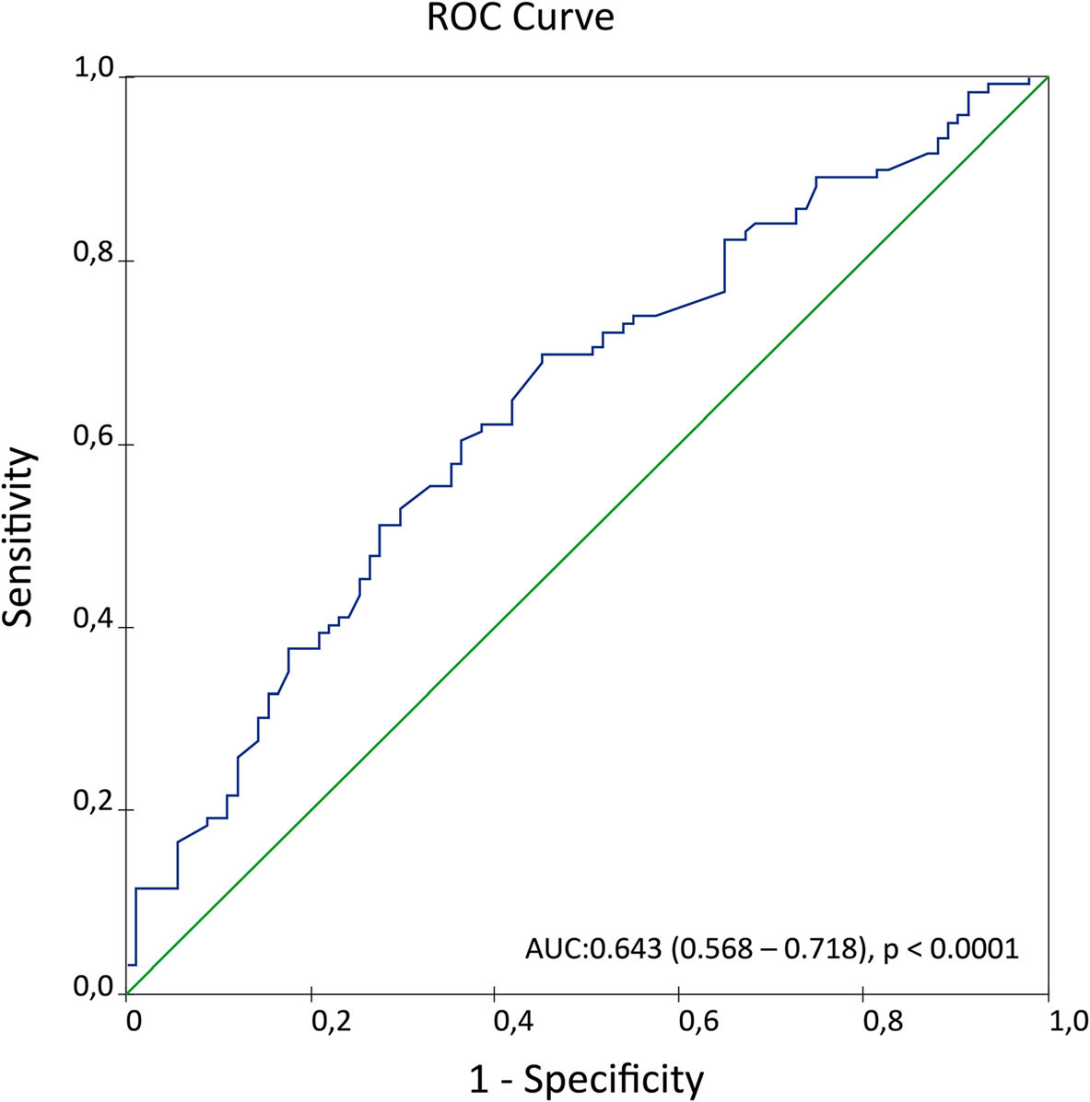
Estimation of mean velocity pulmonary artery (mvPA) optimal threshold by ROC curveto predict the primary combined follow-up endpoint

Optimal cut-off value of mvPA calculated by the ROC curve for the prediction of the primary endpoint was 9 cm/s, [AUC:0.643(0.568–0.718), *p* <  0.0001], Fig. 1**.**

No significant differences in clinical or analytical baseline characteristics according to mvPA values were observed **(Suppl. material** Table 1**).**

### Echocardiography and CMR parameters according to mvPA

sPAP could be estimated by echocardiography in 139(66.2%) individuals and presented similar values in patients with mvPA above and below 9 cm/s whereas TAPSE and the TAPSE/sPAP ratio were lower in patients with reduced mvPA, Table 2**.**

**Table 2:** **Table 2** Baseline characteristics according to mvPA

	mvPA > 9 cm/s *n* = 115	mvPA ≤9 cm/s *n* = 95	Total *n* = 210	*p* value
Echocardiography				
LVEF (%)	28.9 ± 9.8	26.4 ± 9.0	27.8 ± 9.6	0.056
LVEDD (mm)	58.7 ± 7.7	60.1 ± 7.2	59.3 ± 7.5	0.288
LVESD (mm)	46.8 ± 9.2	48.7 ± 8.9	47.6 ± 9.1	0.238
RV diameter (mm)	22.3 ± 5.3	25.4 ± 7.8	23.9 ± 6.9	0.010
TAPSE (mm)	17.8 ± 5.1	16.0 ± 4.2	48.1 ± 14.5	0.044
SPAP (mmHg)^a^	46.8 ± 16.2	49.4 ± 12.7	16.9 ± 4.7	0.321
TAPSE/sPAP ratio (mm/mmHg)^a^	0.45 ± 0.27	0.34 ± 0.15	0.39 ± 0.22	0.020
CMR				
LVEF (%)	28.8 ± 10.4	24.4 ± 9.0	26.9 ± 10.0	0.001
LVEDVI (mL/m^2^)	124.3 ± 41.0	136.2 ± 45.2	129.7 ± 43.2	0.047
LVESVI (mL/m^2^)	91.1 ± 40.2	103.5 ± 43.0	96.7 ± 41.8	0.033
RVEF (%)	46.1 ± 14.6	39.4 ± 16.2	43.1 ± 15.7	0.002
RVEDVI (mL/m^2^)	71.2 ± 27.8	84.1 ± 36.2	77.1 ± 32.5	0.004
RVESVI (mL/m^2^)	39.3 ± 21.4	53.9 ± 32.5	45.9 ± 27.9	< 0.001
LGE ischemic-pattern(n,%)	28(29.8%)	27(36%)	55(32.5%)	0.392
LGE non-ischemic pattern(n,%)	33(35.5%)	35(47.3%)	68(40.7%)	0.123
mvPA (cm/s)	12.7 ± 3.4	7.0 ± 1.6	10.1 ± 3.9	< 0.001
PVR (WU)	3.9 ± 1.7	7.3 ± 1.8	5.5 ± 2.4	< 0.001
RV-PA unit parameters				
Maximal PA-area (cm^2^)	7.9 ± 2.0	9.3 ± 2.5	8.6 ± 2.4	< 0.001
Minimal PA-are a (cm^2^)	5.9 ± 1.8	7.3 ± 1.8	6.6 ± 2.1	< 0.001
PA pulsatility(%)	0.35 ± 0.19	0.30 ± 0.15	0.33 ± 0.18	0.068
RV Emax/Ea	1.03 ± 0.66	0.76 ± 0.52	0.91 ± 0.61	0.002
Right heart catheterisation parameters				
	**mvPA > 9 cm/s** ***n*** **= 27**	**mvPA ≤ 9 cm/s** ***n*** **= 28**	**Total** ***n*** **= 55**	**p value**
mPAP (mmHg)	29.4 ± 11.7	33.4 ± 8.9	31.4 ± 10.5	0.162
PCWP (mmHg)	20.6 ± 8.2	21.1 ± 7.7	20.8 ± 7.8	0.808
RA Pressure (mmHg)	11.1 ± 6.9	13.4 ± 5.9	12.3 ± 6.4	0.243
Cardiac output (l/min)	4.1 ± 1.6	3.2 ± 1.1	3.6 ± 1.4	0.063
PVR (WU)	2.8 ± 2.4	4.1 ± 2.3	3.4 ± 2.4	0.096
TPG (mmHg)	8.5 ± 6.9	12.6 ± 7.4	10.6 ± 7.4	0.042
Pulse Pressure (mmHg)	21.0 ± 9.5	29.6 ± 13.2	25.4 ± 12.9	0.012
PA Compliance (mm^3^/mmHg)	2.02 ± 0.94	1.39 ± 0.79	1.67 ± 0.90	0.022

^a^ Measures available for 139 patients

*CMR* cardiovascular magnetic resonance. *Ea* effective elastance. *Emax* right ventricular maximal end-systolic elastance. *LVEDVI* left ventricular end-diastolic volume index. *LVESV* left ventricular end-systolic volume index. *RVEDV* right ventricular end-diastolic volume index. *RVESV* right ventricular end-systolic volume index. *LGE* late gadolinium enhancement. *LVEF* left ventricular ejection fraction. *LVEDD* left ventricular end-diastolic diameter. *LVESD* left ventricular end-systolic diameter. *MPAP* mean pulmonary artery pressure. *mvPA* mean velocity at the pulmonary artery. *PA* pulmonary artery. *PCWP* pulmonary capillary wedge pressure. *PVR* pulmonary vascular resistance. *RA* right atrium. *RV* right ventricle. *RVEF* right ventricular ejection fraction. *sPAP* systolic pulmonary artery pressure. *TAPSE* tricuspid annular plane excursion. *TPG* transpulmonary gradient. *WU* wood units

In patients with mvPA≤9 cm/s, bi-ventricular function by echocardiography and CMR were significantly reduced and RV diameter and volumes enlarged. Minimal and maximal PA areas and PVR estimated by CMR were increased in patients with mvPA≤9 cm/s as opposed to E_max_/Ea RV-PA coupling ratio, which was significantly reduced in these patients. No differences as to LGE prevalence were observed between both groups.

Mean mvPA values throughout our sample (10.1 ± 3.9 cm/s) were lower than in a sample of healthy controls without structural heart disease that underwent a CMR examination to exclude underlying coronary artery disease or cardiomyopathy (17.3 ± 3.8 cm/s). PVR estimated by CMR was normal in these patients (1.8 ± 1.2), as were RVEF (67.6 ± 7.3%) and RV-PA coupling ratio estimated by E_max_/Ea (2.2 ± 0.77).

### RHC parameters and pulmonary hypertension estimation

PH was confirmed by means of RHC in 39 patients, which accounts for 70.9% of patients who underwent invasive pressure assessment. TPG and PA pulse pressure were greater while PA compliance was lower among patients with mvPA≤9 cm/s, Table 2. No differences in other hemodynamic parameters including mPAP were observed.

### Linear correlation of RV-PA coupling unit parameters

A linear correlation analysis employing the Pearson correlation coefficient between mvPA and other variables reporting on the RV– PA unit coupling was performed, Table 3**.** Linear relation was highest between mvPA and CMR-derived PVR. A statistically significant correlation between mvPA and CMR-derived PA pulsatility, RVEF and Emax/Ea ratio as well as invasive RHC measurements of PA compliance and pulse pressure was observed.

**Table 3:** **Table 3** Linear correlation of RV-PA coupling-unit parameters

CMR-derived variables	Pearson correlation-coefficient	*p*-value
MvPA – PVR (WU)	−0.785	< 0.001
MvPA- Emax/Ea	−0.205	0.003
MvPA – PA pulsatility (%)	+ 0.351	< 0.001
MvPA – RVEF (%)	+ 0.290	< 0.001
**Echocardiographic variables**	**Pearson correlation-coefficient**	**p-value**
mvPA – TAPSE (mm)	+ 0.116	0.211
mvPA – TAPSE/PAPs ratio (mm/mmHg)	+ 0.161	0.133
**RHC variables**	**Pearson correlation-coefficient**	**p-value**
PA compliance (ml/mmHg)	+ 0.455	0.008
Pulse pressure (mmHg)	−0.433	0.004

*CMR* cardiovascular magnetic resonance. *Ea* effective elastance. *Emax* right ventricular maximal end-systolic elastance. *mvPA* mean velocity at the pulmonary artery. *PA* pulmonary artery. *PVR* pulmonary vascular resistance. *RHC* right heart catheterisation. *RVEF* right ventricular ejection fraction. *TAPSE* tricuspid annular plane excursion. *WU* wood units

### Prognosis impact of mvPA estimated by CMR

Univariate analysis for the combined primary endpoint of relevant clinical, analytical as well as echocardiographic and CMR variables is reported in Table 4**.** mvPA ≤9 cm/s predicted adverse events at follow-up, hazard ratio (HR)2.557, 95% confidence interval (CI):1.459–4.482, *p* = 0.001.Other univariate predictors for adverse outcome included a NYHA III-IV/IV functional class, history of previous coronary artery disease, ischemic etiology of cardiomyopathy, diabetes mellitus, stage 3 to 4 chronic renal failure as well as enlarged minimal and maximal PA cross-sectional areas and PVR assessed by CMR.

**Table 4:** **Table 4** Univariate and multivariate Cox regression analysis for the prediction of the primary combined endpoint

Univariate analysis		
Variable	HR(95% CI)	p-value
Clinical variables:		
Sex, male (n,%)	0.832(0.589–1.933)	0.832
Age (years)	1.017(0.994–1.039)	0.147
Arterial hypertension (n,%)	1.171(0.655–2.091)	0.595
Dyslipidemia (n,%)	1.302(0.751–2.258)	0.347
Diabetes mellitus (n,%)	1.793(1.024–3.139)	0.041
Smoker (n,%)	1.391(0.776–2.493)	0.268
NYHA functional class III-IV/IV	2.077(1.508–2.863)	< 0.001
Previous coronary artery disease (n,%)	1.698(0.907–3.180)	0.098
Ischemic vs non-ischemic cardiomyopathy (n,%)	2.226(1.248–3.970)	0.007
Atrial fibrillation (n,%)	1.337(0.737–2.427)	0.339
Stage 3–4 renal failure (eGFR< 50 ml/min/1.73m^2^)	2.224(0.898–5.509)	0.084
Sodium (mEq/L)	0.933(0.852–1.021)	0.131
NT-proBNP (pg/mL)	0.997(0.891–1.011)	0.880
Echocardiography:		
LVEF (%)	0.994(0.965–1.025)	0.711
TAPSE (mm)	0.963(0.886–1.046)	0.370
SPAP (mmHg)	1.014(0.991–1.038)	0.243
TAPSE/SPAP (mm/mmHg)	0.163(0.015–1.791)	0.138
CMR:		
LVEF (%)	0.984(0.957–1.012)	0.265
RVEF (%)	1.007(0.989–1.025)	0.448
LGE presence (n,%)	1.310(0.715–2.398)	0.382
LGE ischemic-pattern (n, %)	1.994(1.036–3.840)	0.039
LGE non-ischemic pattern(n,%)	0.598(0.314–1.139)	0.118
MvPA ≤9 cm/s	2.557(1.459–4.482)	0.001
CMR – PVR (WU)	1.132(1.007–1.272)	0.038
Minimal PA-area (cm2)	1.215(1.043–1.415)	0.012
Maximal PA-area (cm2)	1.180(1.032–1.350)	0.016
PA pulsatility (%)	0.220(0.035–1.382)	0.106
Emax/Ea	1.120(0.716–1.753)	0.620
Multivariate Cox Regression analysis		
Variable	**HR (95% CI)**	**P-Value**
Diabetes mellitus (n,%)	1.261(0.775–2.051)	0.351
NYHA functional class III-IV/IV	2.957(1.841–4.750)	< 0.001
Stage 3–4 renal failure (eGFR< 50 ml/min/1.73m^2^)	2.113(1.144–3.904)	0.017
Ischemic vs non-ischemic cardiomyopathy (n,%)	1.737(1.072–2.816)	0.025
MvPA ≤9 cm/s	1.782(1.105–2.874)	0.018

*CI* confidence interval. *CMR* cardiovascular magnetic resonance. *Ea* effective elastance. *Emax* right ventricular maximal end-systolic elastance. *eGFR* estimated glomerular filtration rate. *HR* hazard ratio. *LGE* late gadolinium enhancement. *LVEF* left ventricular ejection fraction. *mvPA* mean velocity at the pulmonary artery. *NYHA* New York Heart Association. *PVR* pulmonary vascular resistance. *RVEF* right ventricular ejection fraction. *TAPSE* tricuspid annular plane excursion. *WU* wood units

In order to assess whether mvPA acted as an independent prognostic marker, a Cox proportional hazard analysis was performed including all factors that presented with *p* <  0.10 on univariate analysis. The ability of PVR estimated by CMR to predict the combined endpoint was assessed against that of mvPA, to avoid colinearity, as PVR integrates mvPA into its formulae. When both variables were analyzed simultaneously on Cox multivariable regression, only mvPA maintained its prognostic significance and thus, PVR was discarded from the final model. Colinearity was also assessed for other covariates.

On multivariate proportional hazard Cox regression analysis only mvPA≤9 cm/s [HR1.782; 95%CI:1.105–2.874;*p* = 0.018], stage 3–4 renal failure [HR 2.113(95%CI:1.144–3.904);*p* = 0.017], ischemic cardiomyopathy [HR 1.737(95%CI:1.072–2.816);*p* = 0.025] and NYHA functional class III-IV/IV [HR 2.957(95%CI:1.841–4.750);*p* <  0.001] remained statistically significant independent predictors of an adverse outcome.

### Kaplan–Meier survival analysis

**Fig. 2:**
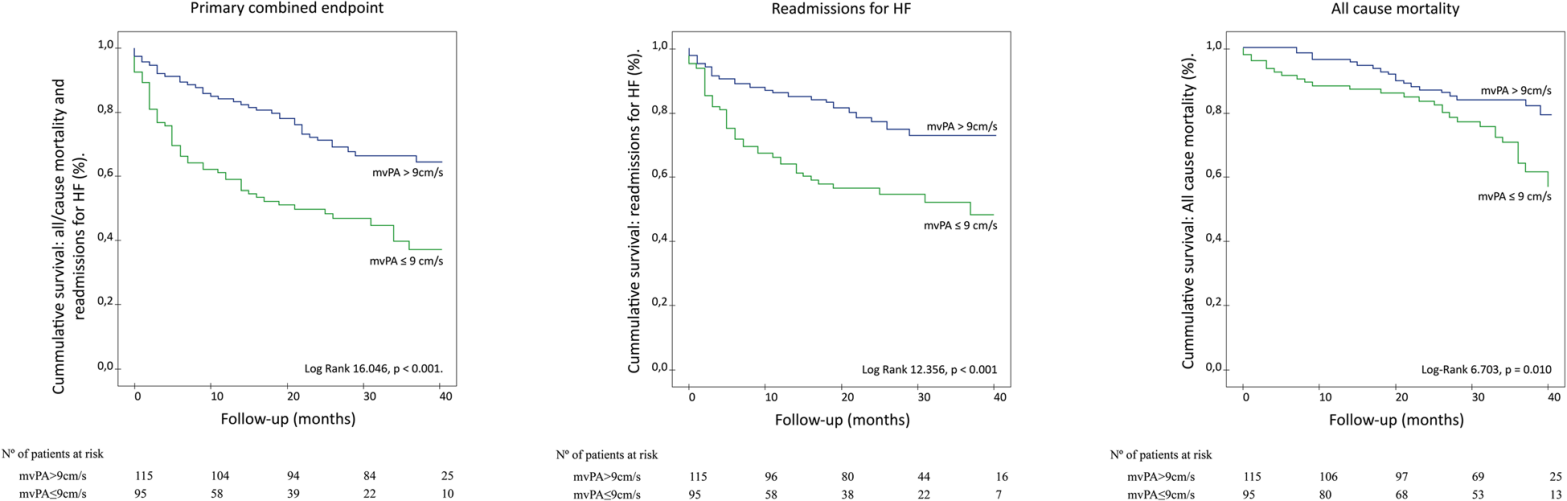
Survival analysis according to mvPA as shown by Kaplan–Meier curves. a. Reduced mvPA≤9 cm/s is associated with higher rates of the primary combined endpoint, b. higher HF readmissions rates, and 2c. higher all-cause mortality rates

A total of 91 patients met the combined primary endpoint, which occurred more frequently in patients with mvPA≤9 cm/s, as indicated in Kaplan-Meier survival curve; Log Rank 16.046, *p* <  0.001;Fig. 2a**.**

When cardiac events were analyzed separately, both HF readmissions (Log Rank Test 12.356, *p* <  0.001;Fig. 2b**)** and all-cause mortality (Log-Rank 6.703, *p* = 0.010;Fig. 2c) were significantly more frequent among patients with mvPA≤9 cm/s.

**Fig. 3:**
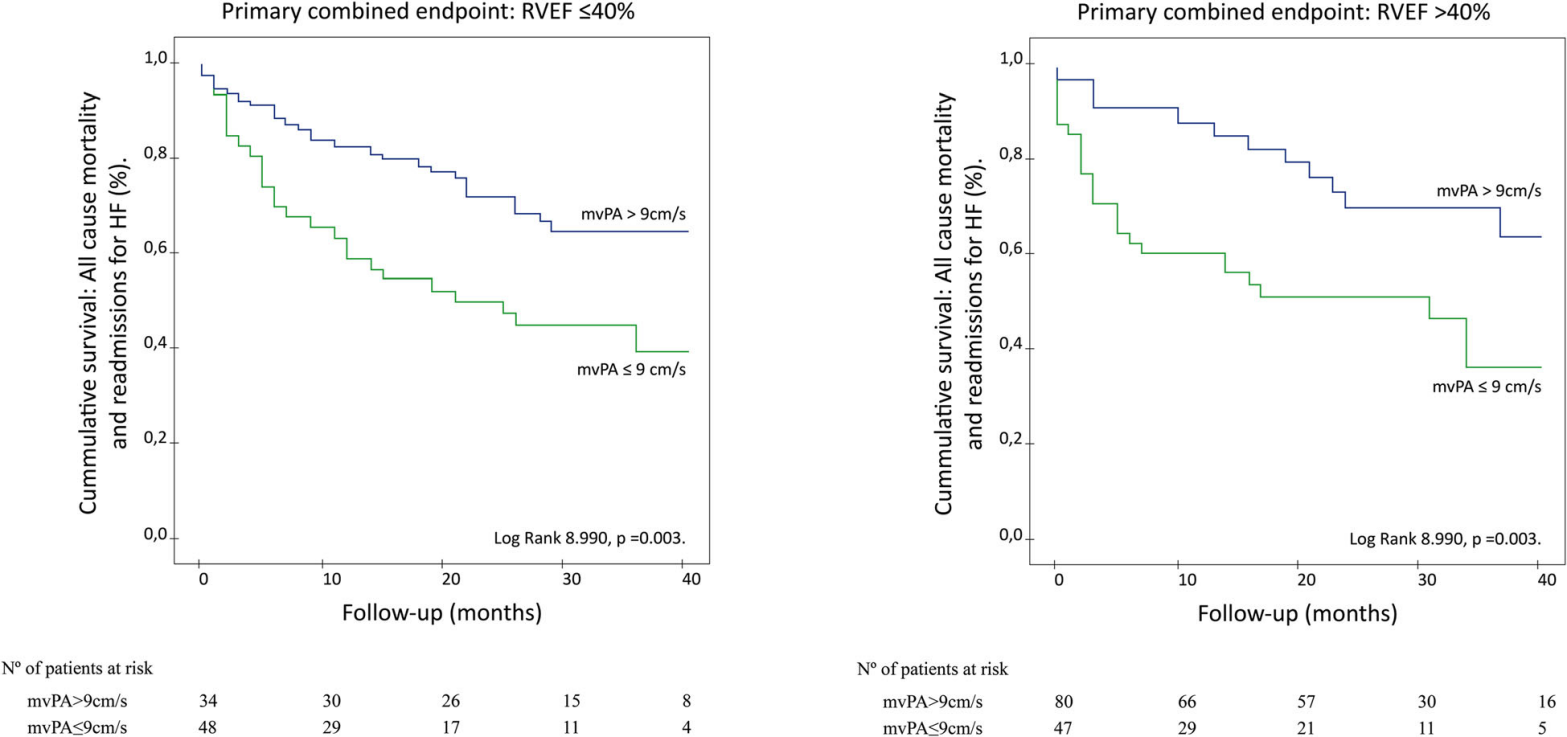
Time to the primary combined endpoint according to mvPA and right ventricular (RV) ejection fraction (EF) as shown by Kaplan–Meier curves. mvPA’s prognostic value remained significant regardless of RV function, considering RVEF≤40% as cut-off value. 3a. Time to primary combined endpoint according to mvPA in patients with RVEF≤40%. 3b. Time to primary combined endpoint according to mvPA in patients with RVEF> 40%

Importantly, prognostic value of mvPA remained significant both in patients with reduced (Log Rank 6.151,*p* = 0.013;Fig. 3a**)** and preserved RV function (Log Rank 8.990, *p* = 0.003;Fig. 3b), considering a cut-off value of RVEF≤40%.

**Fig. 4:**
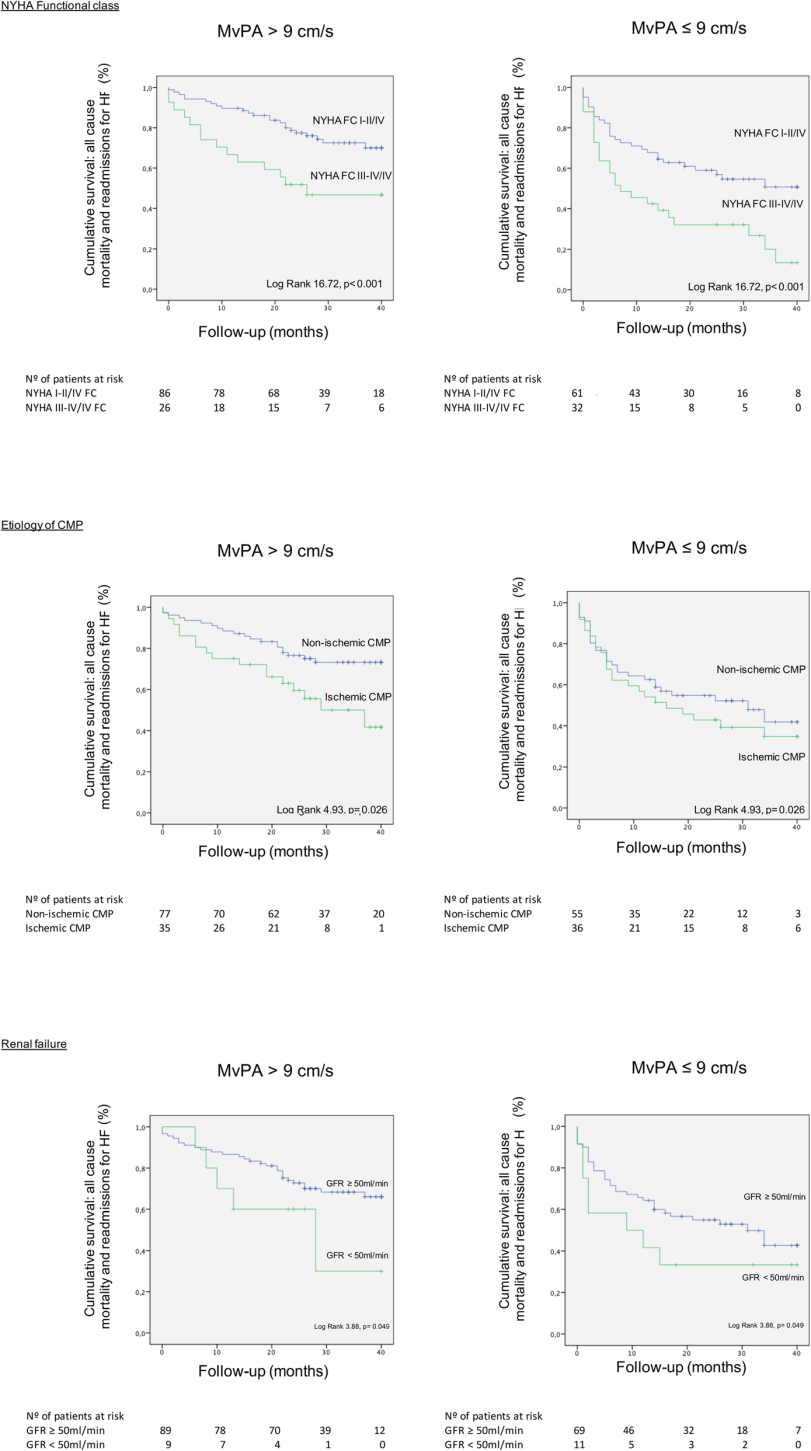
Time to the primary combined endpoint according to mvPA and presence or absence of 4a. NYHA III-IV functional class, 4b. ischemic cardiomyopathy and 4c. advanced renal failure defined as eGFR < 50 ml/min/1.73m^2^. Reduced mvPA≤9 cm/s maintained its prognostic significance regardless of 4a. NYHA functional class, 4b. ischemic etiology of underlying cardiomyopathy and 4c. advanced renal failure

Moreover, when added to Kaplan-Meier survival analysis, mvPA≤9 cm/s improved prognostic accuracy of other known prognostic factors that had been previously identified on Cox analysis. Thus, mvPAmaintained its prognostic significance regardless of NYHA III-IV/IV functional class (Log Rank 16.72, *p* <  0.001;Fig. 4a**)**, ischemic cardiomyopathy (Log Rank 4.93, *p* = 0.026;Fig. 4b**)** or stage 3–4 renal failure (Log Rank 3.88, *p* = 0.049;Fig. 4c**).**

## Discussion

The aim of our study was to evaluate the prognostic value of mvPA as a novel, simple, non-invasive parameter in a cohort of patients with new-onset HFrEF. In addition, we evaluated the potential role of mvPA as a non-invasive surrogate of the RV-PA unit coupling state.

In our sample, individuals with mvPA ≤9 cm/s presented a higher rate of cardiovascular events on follow-up. Reduced mvPA emerged as a robust prognostic indicator as it predicted not only the occurrence of the primary endpoint, but also of its clinical compounds; namely, HF driven readmissions and all-cause mortality, on a separate basis, Fig. 2. The utility of mvPA to predict subsequent HF readmissions has been previously reported in our smaller publication comprising 70 patients also included in this study [20]. The current study adds to prior evidence by identifying reduced mvPA as a predictor for all-cause mortality, while supporting previous results.

Moreover, mvPA ≤9 cm/s emerged as an independent prognostic indicator in multivariate Cox regression analysis for the prediction of the primary combined endpoint, together with NYHA III-IV/IV functional class, ischemic cardiomyopathy and stage 3–4 renal failure, Table 4. Importantly, mvPA≤9 cm/s improved prognostic accuracy for the detection of the primary endpoint when included in Kaplan-Meier analysis over the above-mentioned prognostic predictors in HF, i.e. NYHA III-IV/IV functional class, ischemic cardiomyopathy and stage 3–4 renal failure**,** Fig. 4**.**

Over the last years, several studies have described the prognostic value of PVR estimated by CMR in the assessment of HF with suspected PH [26, 27]. However, in HF patients, PA compliance and stiffness are altered in early disease stages, before significant rise in PVR occurs [10–14]. Reduced PA compliance and PA stiffness result in inefficient RV-PA coupling [7–9] and have emerged as strong, early prognostic predictors in this setting [18, 19, 28].

Based on this evidence, we decided to study the potential role of mvPA as a surrogate marker of the RV-PA unit coupling state, as mvPA combines information on RV function and RV afterload. We observed that patients with mvPA below the cut-off value of 9 cm/s presented worse PA-RV coupling, as indicated by reduced Emax/Ea and TAPSE/SPAP ratios, reduced PA compliance and larger PA diameters, Table 2. Altogether, these data support the role of mvPA in the assessment of the RV-PA coupling unit.

Of note, mvPA outperformed aforementioned RV-PA coupling indicators in our sample. TAPSE/SPAP ratio is based on echocardiographic determination of RV function and estimation of sPAP. Both echocardiographic determinations are less accurate than RVEF measured by CMR and quantification of PA pressures by RHC, which justifies its lower prognostic value. On the other hand, while Emax/Ea does rely on CMR to measure RV volumes, this formula does not take into account the effect of PCWP on arterial load calculations [29], which could reduce its precision when applied to HF, as opposed to patients with pulmonary arterial hypertension.

Impairment of the RV-PA coupling unit in patients with mvPA ≤9 cm/s was one of the underlying mechanisms that justified a worse outcome in these subjects. Also, patients with reduced mvPA presented lower bi-ventricular function on echocardiography and CMR and reduced cardiac output by RHC, which are major determinants of a poorer outcome in HF.

Interestingly, mvPA≤9 cm/s predicted worse outcome both in patients with normal and reduced RV function, Fig. 3. As opposed to other RV-PA coupling indicators such as Emax/Ea or the TAPSE/SPAP ratio as well as PVR estimated by CMR, mvPA is not directly calculated from RVEF or RV stroke volume. Although patients with mvPA≤9 cm/s presented lower mean RVEF in our sample, mvPA allowed for further prognostic stratification both in patients with and without associated RV dysfunction.

Significantly, patients with mvPA≤9 cm/s and preserved RV function presented a worse outcome than patients with associated RV dysfunction but mvPA> 9 cm/s, Fig. 3. This finding is of great importance in HFrEF, especially in those cases with associated PH, as it enables identification of patients at higher risk of subsequent cardiovascular events before development of RV dysfunction, which constitutes an end-stage event [1–3].

Management of patients with mvPA ≤9 cm/s should be based on optimal guideline-directed HF treatment. Use of drugs aiming to reduce RV and pulmonary pressures and close monitoring of RV function appear as reasonable strategies. Whether specific additional interventions may provide prognostic benefit in these patients remains an open research question to date, that should be assessed in future trials.

The fact that mvPA can be estimated in a complete non-invasive manner with CMR makes it a specially attractive technique in the setting of HF. CMR is often performed as part of the diagnostic work-up in HFrEF as this technique enables not only a morpho-functional assessment of cardiac chambers, but also a diagnostic approach to the underlying HF etiology, by characterization of myocardial tissue. Indeed, LGE assessment is a recognized prognostic indicator in HF [30]. In our sample, an ischemic LGE pattern on CMR was more frequently observed in patients presenting with the primary combined endpoint and ischemic cardiomyopathy constituted an independent prognostic factor. Neither non-ischemic LGE pattern nor fibrosis amount were related to a worse outcome in our study.

Quantification of mvPA is relatively simple and non-operator dependant, as most measures are automatized, and calculations performed through dedicated software. Thus, it can easily be included in a routine CMR study with a mild extension of 4–6 min including examination and post-processing time. MvPA determination in patients undergoing a CMR examination appears as an appealing option for risk stratification and assessment of the RV-PA unit and could be incorporated into clinical practice if its utility is confirmed in larger samples. Importantly, appropriate sequences must be selected in AF patients with significant R-R interval variability to ensure accurate results.

### Limitations

The main limitation of this study is the reduced availability of RHC data in our sample. Being RHC an invasive, clinically driven study, it was only performed in a small subset of patients, *n* = 55(25.7%), at the discretion of the patient’s physician. Further studies including more patients undergoing RHC should evaluate the prognostic value of mvPA measured by CMR in HFrEF, with and without associated PH. Lack of a validation cohort for mvPA’s cut-off value of 9 cm/s as a predictor of worse outcome is another significant limitation.

## Conclusions

mvPA is a simple, non-invasive parameter that can be easily determined by CMR. In a cohort of new-onset HFrEF patients with and without associated PH, we identified mvPA≤9 cm/s as an independent prognostic indicator on multivariate Cox regression together with NYHA III-IV/IV functional class, ischemic cardiomyopathy and stage 3–4 renal failure. Patients with mvPA≤9 cm/s presented worse outcome irrespective of RV function. Thereby, assessment of mvPA has the potential to identify a higher-risk population before structural damage onset. Moreover, mvPA emerged as a non-invasive surrogate marker of the RV-PA coupling state in our sample.

## Supplementary information

**Additional file 1: Supplementary material.** intended for publication as an online data supplement. Suppl. Table 1**.** Baseline characteristics according to mvPA values.

## Abbreviations

AF: Atrial fibrillation
AUC: Area under the curve
bSSFP: Balanced steady state free precession
CMR: Cardiovascular magnetic resonance
Ea: Effective elastance
ECG: Electrocardiography
eGFR: Estimated glomerular filtration rate
Emax: Maximal end-systolic elastance
ESV: End-systolic volume
HF: Heart failure
HFmEF: Heart failure with intermediate ejection fraction
HFrEF: Heart failure with reduced ejection fraction
LGE: Late gadolinium enhancement
LV: Left ventricle/left ventricular
LVEF: Left ventricular ejection fraction
mPAP: Mean pulmonary artery pressure
mvPA: Mean velocity at the pulmonary artery
NYHA: New York Heart Association
PA: Pulmonary artery
PCWP: Pulmonary capillary wedge pressure
PH: Pulmonary hypertension
PVR: Pulmonary vascular resistance
RHC: Right heart catheterization
RV: Right ventricle/right ventricular
RVEF: Right ventricular ejection fraction
sPAP: Systolic pulmonary artery pressure
SV: Stroke volume
TAPSE: Tricuspid annular plane systolic excursion
TPG: Transpulmonary pressure gradient
