# Supplementary material for: Prognostic value of mean velocity at the pulmonary artery estimated by cardiovascular magnetic resonance as a prognostic predictor in a cohort of patients with new-onset heart failure with reduced ejection fraction
Source: J Cardiovasc Magn Reson. 2020 Apr 30;22:28. doi: 10.1186/s12968-020-00621-3 (PMC7191770; doi:10.1186/s12968-020-00621-3)
Supplement: Supplementary file 1 — Additional file 1: Supplementary material. intended for publication as an online data supplement. Suppl. Table 1. Baseline characteristics according to mvPA values. [file 12968_2020_621_MOESM1_ESM.docx]

**Supplementary material:** intended for publication as an online data supplement.

**Suppl. Table 1.** Baseline characteristics according to mvPA values.

|  | mvPA > 9 cm/s  n = 115 | mvPA ≤ 9 cm/s  n = 95 | Total  n = 210 | p value |
| --- | --- | --- | --- | --- |
| Age | 64.2 ± 11.1 | 63.9 ± 14.2 | 64.1 ± 12.6 | 0.908 |
| Sex, male (n,%) | 85 (73.9%) | 62 (65.3%) | 147 (70-0%) | 0.173 |
| Arterial hypertension (n,%) | 78 (68.4%) | 58 (61.7%) | 136 (65.4%) | 0.386 |
| Diabetes Mellitus (n,%) | 41 (36.0%) | 45 (47.9%) | 89 (41.3%) | 0.083 |
| Dyslipidaemia (n,%) | 55 (48.2%) | 47 (50.0%) | 102 (49%) | 0.801 |
| Atrial fibrillation (n,%) | 34 (29.6%) | 28 (29.5%) | 62 (29.5%) | 0.988 |
| Implanted cardiodefibrillator (n,%) | 41(35.6%) | 36 (37.8%) | 44(21.6%) | 0.411 |
| Cardiac resynchronization therapy (n,%) | 7(6.1%) | 3 (3.2%) | 10(4.8%) | 0.256 |
| Coronary artery disease (n,%) | 27 (23.5%) | 26 (27.7%) | 53 (25.4%) | 0.489 |
| eGFR (ml/min/1.73m^2^) | 74.2 ± 17.8 | 72.9 ± 18.2 | 73.7 ± 17.9 | 0.705 |
| Stage 3-4 renal failure  (eGFR < 50 ml/min/1.73m^2^) | 10 (10.0%) | 12 (14.6%) | 22 (12.1%) | 0.340 |
| Sodium (mEq/L) | 138.2 ± 3.1 | 137.2 ± 3.6 | 137.7 ± 3.3 | 0.063 |
| NT-proBNP | 5526 ± 5545 | 6281 ± 4006 | 5953 ± 4821 | 0.522 |
| NYHA Functional Class |  |  |  | 0.077 |
| I | 21 (18.3) | 12 (12.6%) | 33 (15.7%) |  |
| II | 68 (59.1%) | 49 (51.6%) | 117 (55.7%) |  |
| III | 22 (19.1%) | 25 (26.3%) | 47 (22.4%) |  |
| IV | 4 (3.5%) | 9 (9.5%) | 13 (6.2%) |  |

GFR = glomerular filtration rate. MvPA = mean velocity at the pulmonary artery. Nt-proBNP = N terminal brain natriuretic type peptide. NYHA = New York Heart Association.
